# Antioxidant capacities and non-volatile metabolites changes after solid-state fermentation of soybean using oyster mushroom (*Pleurotus ostreatus*) mycelium

**DOI:** 10.3389/fnut.2024.1509341

**Published:** 2024-12-06

**Authors:** Mengxin He, Qing Peng, Xiaoqing Xu, Bo Shi, Yu Qiao

**Affiliations:** Feed Research Institute, Chinese Academy of Agricultural Sciences, Beijing, China

**Keywords:** *Pleurotus ostreatus*, solid-state fermentation, soybean, antioxidant activity, metabolome

## Abstract

Given the abundance of beneficial properties and enzymes secreted by edible oyster mushrooms, their mycelium could serve as a starter for fermented foods to enhance their nutritional and bioactive quality. This study aimed to investigate the effects on the nutritional ingredients, antioxidant activity, and non-volatile metabolites during solid-state fermentation (SSF) of soybeans by *Pleurotus ostreatus* mycelium. The results indicated that the contents of dietary fiber and starch in fermented soybeans decreased, while the amounts of protein and lipid increased after SSF (*P* < 0.05). Analysis of the total phenolic content (TPC) and antioxidant activities of the fermented soybeans revealed that the methanolic extracts significantly increased TPC and antioxidant activities against intracellular reactive oxygen species (ROS) in lipopolysaccharide (LPS)-stimulated RAW 264.7 macrophages, as well as against DPPH and ABTS radicals *in vitro*. A total 154 differential metabolites were identified after SSF, and a Spearman correlation study revealed a direct relationship between antioxidant activities and certain metabolites including phenolic compounds, oligopeptides, and free fatty acids etc. Among these metabolites, phenolic compounds produced by the shikimic acid pathway were diverse in variety and had the greatest multiple differences. The study discovered that a potential mechanism involving SSF with *P. ostreatus* mycelium increased the antioxidant activity of soybeans.

## 1 Introduction

Soybeans (*Glycine max* L.) are the main crop grown among legumes and rank the second-largest food crop in the world after cereals. After years of expansion, soybeans have become a mainstay of Southeast Asian cooking, and their particular characteristics have attracted the attention of the food industry (1, 2). Because of their nutritional qualities and useful components, soybeans are regarded as one of the best meat alternatives (3–5). Furthermore, numerous epidemiological studies have demonstrated the advantages of soybeans in lowering the prevalence of heart problems, menopausal symptoms, and cancers such as prostate, breast, and colon cancers (5–9).

A great option for meeting individual increasing needs for healthy, tasty, and sustainably produced food is fermented soybean (10–12). Multiple complex processes, including microbial metabolism, enzyme catalysis, lipid oxidation, amino acid breakdown, and Maillard browning, are involved in the fermentation of soybeans (13–15). Trypsin inhibitors, which prevent the absorption of nutrients from food, are efficiently eliminated from soybeans during fermentation, reducing their antinutritional effects (16, 17). Certain vitamin contents, such as vitamin K_2_ and vitamin B_12_, noticeably increase in fermented soybean products (12, 18). Fermentation breaks down phytates that bind with minerals, making iron and copper more bioavailable (19). Through fermentation, isoflavone' glycoside forms are changed into aglycone forms, increasing its bioavailability (20).

The oxidative stress caused by reactive oxygen species (ROS) has been linked to the development of multiple chronic diseases, such as rheumatoid arthritis, diabetes mellitus, neurological disorders, and cataract formation (21). It has been discovered that the soybean fermentation produces significant antioxidant activity in both primary and secondary metabolites including, free amino acids, small peptides, flavonoids, phenolic acids and phytosterols, etc. (3, 10). Furthermore, by increasing the activity of antioxidant enzymes such as superoxide dismutase (SOD), catalase (CAT), and glutathione peroxidase (GSH-Px), soybeans through fungal solid-state fermentation demonstrated a preventive effect on lipid peroxidation in the liver of cholesterol-fed rats (22). Stronger antioxidant activity was found in fermented soybean foods made with *B. subtilis* GD1, *B. subtilis* N4, *B. velezensis* GZ1, *L. delbrueckii* subsp. bulgaricus, and *Hansenula anomala* (23). Various types of microbes, such as filamentous fungi including *Aspergillus* spp., *Mucor* spp. and *Rhizopus* spp., bacteria including *Bacillus* spp., and lactic acid bacteria are involved in soybeans fermentation (3, 10). Mushroom mycelium grows quickly and is also used as a starter for fermenting soybeans. After 20 days of fermentation with *Irpex lacteus* mycelia, soybeans exhibited ~4–5 times more total phenolic, flavonoid, isoflavone, and 2,2-diphenyl-1-picrylhydrazyl (DPPH) radical scavenging (24). The fermentation process changed the physical characteristics of soybean and enhanced their antioxidant qualities, which could be affected by the choice of microbial species, temperature, duration, and concentration of salt and environmental factors (3, 14, 25). Phenolic compounds were found to be the major contributor to the antioxidant effects in fermented soybeans (3). β-glucosidase convert isoflavone glucosides to isoflavone aglycones and other metabolites during the mold-fermented soybean process. It has been proposed that isoflavone aglycones have a greater antioxidant capability than their glycoside forms (3, 26). However, due to the decreased β-glucosidase activity, isoflavone aglycones had very minor effects on the antioxidant activity of the bacterially fermented soybean. Small peptides and free amino acids particularly those made of hydrophobic, aromatic, acidic, and basic amino acids accumulated as a result of the high activity of proteases in bacterial fermented soybean, which were strongly correlated with the antioxidant activity (27). Microorganisms convert substances into bioactive compounds that are associated with antioxidant activity through enzymatic processes (13). Thus, it is crucial to use novel microorganisms with special enzyme-producing capabilities to create new functional fermented foods.

*Pleurotus ostreatus*, also known as oyster mushroom, is a white-rot macrofungus that secretes a variety of enzymes, particularly lignocellulolytic enzymes, throughout its growth (28). Its chemical composition consists of polysaccharides (β-glucans), dietary fibers, unsaturated fatty acids, peptides, terpenoids, sterols, and physiologically active proteins such as lectins and enzymes (29). Numerous studies have documented the bioactive effects of compounds derived from *P. ostreatus*. Two novel sesterterpenes and two new triterpenes that demonstrated antiprotozoal action against *Trypanosoma cruzi* and *Plasmodium falciparum* were identified by Annang et al. (30). From the methanolic extracts of *P. ostreatus*, the coumarins 5-methoxy-(E)-subodiene and toddaculin were isolated, as well as the steroid ergosterol, which showed notable antifungal activity against *Penicillium digitatum* (31). Three novel amino acid compounds with antifungal activity against *Candida albicans* were isolated from the ethyl acetate extracts of *P. ostreatus*' solid culture (32). Furthermore, 3-(2-aminophenylthio)-3-hydroxypropanoic acid from *P. ostreatus* aqueous extracts showed potent antibacterial and antifungal properties (33).

While some research has been done on the use of oyster mushrooms to increase the nutritional and health-promoting properties of food products (29, 34, 35), there have been no studies conducted on the use of *P. ostreatus* mycelium to improve the quality of soybean products. To enhance the antioxidant properties of soybeans, our research focused on the potential effects of novel fermented soybeans using oyster mushroom with strong antioxidant activity. The objective of these investigations was to ascertain the alterations in the non-volatile metabolites and antioxidant capacity during the fermentation of soybeans with *P. ostreatus*. The findings of this study could provide a theoretical foundation for further research and commercial development, such as fermenting soybeans with edible mushrooms.

## 2 Materials and methods

### 2.1 Materials

Soybeans (*Glycine max* L.) were sourced from Mudanjiang City, Heilongjiang Province, China. *Pleurotus ostreatus* (CGMCC 5.784) was purchased from the China General Microbiological Culture Collection Center (CGMCC) and stored in potato dextrose agar (PDA) slant medium at 4°C.

DPPH was purchased from Aladdin Biochemical Technology Co., Ltd (Shanghai, China). Gallic acid, Folin-Ciocalteu's phenol reagent and 2,2′-Azinobis-(3-ethylbenzthiazoline-6-sulphonate) (ABTS) were purchased from Sigma Chemical Co. (St. Louis, MO, USA). 6-hydroxy-2,5,7,8-tetramethylchroman-2-carboxylic acid (Trolox) was purchased from Yuanye Bio-Technology Co., Ltd (Shanghai, China). RAW 264.7 macrophages were purchased from Beijing Dingguo Changsheng Biotechnology Co., Ltd (Beijing, China). All other chemicals and solvents were of analytical reagent grade and acquired from Sinopharm Chemical Reagent Co., Ltd. (Shanghai, China).

### 2.2 Methods

#### 2.2.1 Preparation of fermented soybeans by P. ostreatus mycelium

The soybeans were subjected to solid state fermentation (SSF) according to the study by Zhai et al. (36) with slight modifications. The slant mycelia of *P. ostreatus* were transferred onto petri plates containing a broth made of 0.4% PDA for cultivation at 25°C for ~10 days. Soybeans were soaked in deionized water (1:5, w/v) for 8 h at room temperature, and the moisture content of soybeans was about 60%. Tissue culture bottles (80 × 90 mm) were filled with 120 g of soaked soybeans and sterilized by autoclaving at 121°C for 90 min. Three tiny mycelia of 1 cm × 1 cm from a fully colonized petri dish cultured on PDA were added to the surface of the cooled soybeans as an inoculant. The bottles were then incubated at 25°C and 70% humidity in the dark until the fungal mycelia covered the entire bottle (33 days). The soybeans used as the control underwent the same procedure without being inoculated.

#### 2.2.2 Assay of general composition of soybeans

Both the unfermented soybeans (USB) and fermented soybeans (FSB) were heat-dried at 60°C for 24 h until they achieved a constant weight after the culture. After that, the material was ground using a grinder with a Ø40 mm diameter for further analysis. The unfermented and fermented soybeans were analyzed for their chemical composition. The content of moisture, crude protein, reducing sugars (37), starch (38), total dietary fiber, soluble dietary fiber, and insoluble dietary fiber (39) were determined according to standard methods described by the Association of Official Analytical Chemists (AOAC). Lipid content was determined using the method reported by Idamokoro et al. (40). All determinations were performed in triplicate, and the mean values with standard deviations were calculated and reported.

#### 2.2.3 Assay of the total phenolic content

Determining the total phenolic content (TPC) was conducted by the method described by Donlao and Ogawa (41). Dried and crushed samples after fermentation were homogenized with 80% (v/v) methanol (10 g sample/200 mL 80% MeOH w/v). Afterward, the extract was filtered and collected in a dark glass bottle. Briefly, 20 μL of the diluted extract was mixed with 20 μL of 50% Folin-Ciocalteau reagent in 96-well plates and kept in the dark. After a 30 min interval, 40 μL of 7.5% Na_2_CO_3_ and 120 μL of deionized water were added. After letting the mixture sit in the dark for 2 h, the SYNERGY H1 microplate reader (Biotek Instruments, Inc. USA) was used to measure the absorbance at 765 nm. The results were expressed as milligrams of gallic acid equivalent per g (mg GAE/g).

#### 2.2.4 Determination of antioxidant activity

The supernatant of methanolic extracts was concentrated and lyophilized, then prepared into different concentrations (ranging from 0.5 to 16 mg/mL) with 80% (v/v) methanol to determine antioxidant activity. DPPH radical scavenging activity was measured according to the method reported by Sanjukta et al. (42). Briefly, 100 μL of the supernatant of methanolic extracts, Trolox and diluted methanolic extracts (0.5, 1, 2, 4, 8 and 16 mg/mL) were mixed with DPPH-methanol (0.2 mM, 100 μL) in a 96-well plate. The mixture was kept in the dark for 30 min at room temperature and the absorbance at 517 nm was read. 80% (v/v) methanol was used instead of the samples for the blank control. DPPH scavenging activity was determined as follows: DPPH scavenging activity = (1-A_s_/A_b_) ×100%, where A_b_ is the absorbance of the 80% (v/v) methanol reacted with DPPH, and A_s_ is the absorbance of the different samples reacted with DPPH. The calibration curve was drawn with different concentrations of Trolox as the horizontal coordinate (x) and DPPH scavenging activity as the vertical coordinate (y) (y = 0.0131x + 0.0067; R^2^ = 0.9988). The Trolox calibration curve (0 ~ 70 μmol/L) was used to quantify antioxidant activity allowing for the expression of the ability to radical scavenging activity of unfermented and fermented soybeans as μmol Trolox equivalents per gram.

The scavenging activities of samples against ABTS were determined according to the method reported by Ketnawa and Ogawa (43). ABTS solution (7.00 mmol/L) and K_2_S_2_O_8_ solution (4.90 mmol/L) were mixed in a 2:1 (v: v) ratio and kept in the dark for 16 h. Subsequently, deionized water was added to the mixture until the absorbance at 734 nm was 0.7 ± 0.02. The mixture was used as the ABTS working solution. 40 μL of the supernatant of methanolic extracts, Trolox and diluted methanolic extracts (0.5, 1, 2, 4, 8 and 16 mg/mL) was added to 200 μL of ABTS working solution. The mixture was incubated in the dark at room temperature for 5 min and the absorbance at 734 nm was read. 80% (v/v) methanol was used instead of the samples for the blank control. ABTS scavenging activity was determined as follows: ABTS scavenging activity = (1-A_s_/A_b_) ×100%, where A_b_ is the absorbance of the 80% (v/v) methanol reacted with ABTS, and As is the absorbance of the different samples reacted with ABTS. The calibration curve was drawn with different concentrations of Trolox as the horizontal coordinate (x) and ABTS scavenging activity as the vertical coordinate (y) (y = 0.0056x + 0.0058; R^2^ = 0.9995). The Trolox calibration curve (0 ~ 160 μmol/L) was used to quantify antioxidant activity allowing for the expression of the ability to radical scavenging activity of unfermented and fermented soybeans as μmol Trolox equivalents per gram.

Intracellular reactive oxygen species (ROS) scavenging activity was assayed according to Xu et al. (44) in lipopolysaccharide (LPS)-stimulated RAW 264.7 macrophages with modifications. RAW 264.7 macrophages were inoculated (25,000 cells/well) in 96-well plates and allowed to develop for 24 h. Cells were then exposed to 1.5 μL of unfermented and fermented soybeans (ranging from 0 to 40 μg/mL) and 200 μL of culture media including 1 μL/mL LPS for 24 h. As a negative control, cell culture media without soybeans was added. After incubation, each well received 200 μL of 2′,7′-dichlorofluorescein diacetate DCFH-DA solution (10 μM), which was then incubated for 30 min at 37°C in an incubator with 5% CO_2_. Fluorescence intensities of each well were measured at 485 and 528 nm using a Synergy H_1_ microplate reader (BioTek, USA). The percentage generation of ROS in comparison to the negative control was calculated based on the value of F485/528. ROS% = (The value of sample)/(The value of negative control) ×100%. After fluorescence intensity evaluation, the dye was disposed of and the cells were rinsed twice with ice-cold PBS. Subsequently, fluorescence images were rapidly captured using a green fluorescent protein (GFP) channel at a 20 × magnification on an EVOS M7000 system (Thermo Fisher Scientific, Waltham, MA).

#### 2.2.5 Analysis of metabolites

To extract metabolites, 50 mg of sample and 400 μL of 80% (v/v) methanol containing 0.02 mg/mL of an internal standard (L-2-chlorophenylalanine) were added to a 1.5 mL centrifuge tube (45). After 30 s of vortex mixing, the samples were sonicated for 30 min at 5°C. Subsequently, the samples were kept at −20°C for 30 min to precipitate the proteins. The materials were then centrifuged for 15 min at 4°C and 13,000 g and the supernatant was collected for non-targeted metabolome analysis (46). A quality control (QC) sample was prepared by mixing all samples to be a pooled sample.

The LC-MS/MS analysis was conducted using the SCIEX UPLC-Triple TOF 5600 system with an ACQUITY HSS T3 column (100 mm × 2.1 mm i.d., 1.8 μm; Waters, USA) at Majorbio Bio-Pharm Technology Co. Ltd. (Shanghai, China). The injection volume was 10 μL. The mobile phases comprised 0.1% formic acid in water: acetonitrile (95:5, v/v) (solvent A) and 0.1% formic acid in acetonitrile: isopropanol: water (47.5:47.5:5, v/v/v) (solvent B). The flow rate was 0.40 mL/min, and the column temperature was maintained at 40°C. The Ultra Performance Liquid Chromatography (UPLC) system was coupled to a quadrupole-time-of-flight mass spectrometer (Triple TOFTM 5600+, Sciex, USA) equipped with an electrospray ionization (ESI) source operating in both positive and negative modes. The optimal conditions were set as follows: source temperature at 550°C; curtain gas (CUR) at 30 psi; Ion Source Gas1 and Gas2 at 50 psi each; ion-spray voltage floating (ISVF) at −4,000V in negative mode and 5,000 V in positive mode; declustering potential at 80 V; collision energy (CE) set to 20–60 eV rolling for MS/MS. Data acquisition was performed using the Information Dependent Acquisition (IDA) mode, and detection was carried out over a mass range of 50–1,000 m/z.

#### 2.2.6 Statistical analysis

All results of experiments conducted in triplicate were presented as mean ± standard deviation (SD). Data were analyzed using SPSS software (Inc., Chicago, IL, USA) employing one-way ANOVA and T-test with a confidence interval of 95 % (P < 0.05) for means. Regular graphs and tables were made in excel. The graphs in metabolomics were made through the free online platform of majorbio choud platform (www.cloud.majorbio.com).

Principal component analysis (PCA) was used to determine the degree of metabolite difference between different samples and the degree of difference within groups. The difference of specific metabolites before and after fermentation was observed through the analysis of metabolite cluster heat map. Orthogonal partial least squares discriminant analysis (OPLS-DA) was carried out to determine the differences in non-volatile metabolites between unfermented and fermented soybeans. Variable importance in the projection (VIP) ≥ 1, fold change (FC) ≥ 2 or ≤ 0.5 and P < 0.001 were used as screening conditions to identify differential metabolites. Spearman correlation analysis was performed for the difference metabolites and antioxidant activity after SSF.

## 3 Results and discussion

### 3.1 Basic characteristic of fermented soybeans by *P. ostreatus*

*Pleurotus* species have the ability to extensively degrade a wide variety of substrates to release small molecules from carbohydrates and other macromolecular nutrients of soybeans. This is achieved by secreting the requisite extracellular oxidative (ligninolytic) and hydrolytic (cellulases and hemicellulases) enzymes to liberate low-molecular-weight molecules that can be absorbed for their nourishment (47). The soybeans were inoculated with mycelia of *P. ostreatus*, and cultured at 25 ± 0.1°C. The mycelium grew slowly and filled the entire bottle within 33 days. The general composition of fermented soybeans by *P. ostreatus* is shown in Table 1. Compared to the unfermented soybeans, the starch and dietary fiber (DF, including soluble and insoluble dietary fiber) in fermented soybeans were consumed during the growth of *P. ostreatus* mycelia, reducing the starch and DF content from 16.62% to 11.23% and from 19.51% to 12.8%, respectively. Consequently, the reduced sugar content increased from 0.94% to 3.97%. Oyster mushrooms are capable of enzymatically breaking down a variety of substrates that comprise dietary fiber and starch into low molecular weight soluble chemicals. Amylase is the enzyme that breaks down starch, while cellulase, xylanase, and laccase are the three main groups that break down dietary fiber. Lim et al. investigated the activities of amylase (EC 3.2.1.1), cellulase (EC 3.2.1.4), laccase (EC 1.10.3.2), and xylanase (EC 3.2.1.8) in discarded *P. ostreatus* mushroom compost. The results showed that amylase, cellulase, xylanase, and laccases activity were 2.97 U/g, 1.67 U/g, 91.56 U/g, and 2.97 U/g, respectively (48). Since the biomass of *P. ostreatus* increased throughout growth, the fermented soybeans' crude protein and lipid contents increased by 27.80% and 5.0%, respectively, compared to the unfermented soybeans, reaching 45.33% and 20.34% after fermentation (Table 1).

**Table 1:** General composition indicators in unfermented soybeans (USB) and fermented soybeans (FSB)^1^.

**Item (%)**	**USB**	**FSB**
Protein	35.47 ± 0.14^b^	45.33 ± 0.11^a^
Lipid	19.38 ± 0.31^b^	20.34 ± 0.28^a^
Soluble dietary fiber	5.64 ± 0.05^a^	2.82 ± 0.09^b^
Insoluble dietary fiber	13.87 ± 0.15^a^	9.98 ± 0.11^b^
Reducing sugar	0.94 ± 0.00^b^	3.97 ± 0.07^a^
Starch	16.62 ± 0.15^a^	11.23 ± 0.16^b^

^1^Different lowercase letters on the same line were significantly different (P < 0.05).

Certain solid fermented products showed comparable tendencies. Zhai et al. (36) found that adding *Agaricus blazei* to wheat, rice, oats, maize, millet, broomcorn millet, and sorghum during fermentation increased the amount of reducing sugar by 100.77, 7.12, 12.35, 10.85, 57.55, and 58.68 times, respectively, compared to the control. The solid-state fermentation of tempeh resulted in a 21.7% increase in protein content as well as an increase in essential amino acids content, particularly amino acids containing sulfur (49).

**Figure 1:**
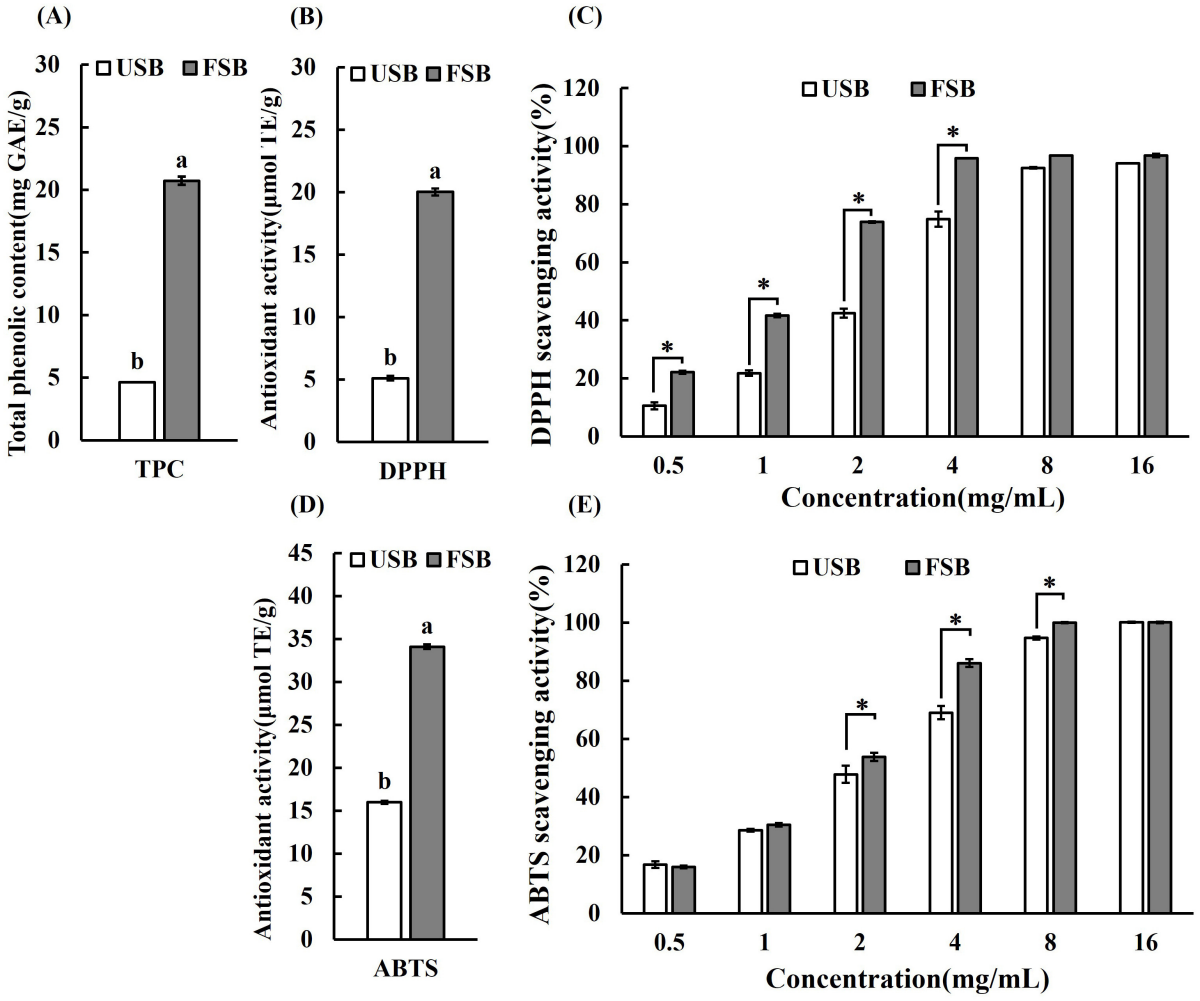
The total phenolic contents and the antioxidant capacity of unfermented soybeans (USB) and fermented soybeans (FSB) by P. ostreatus mycelium. **(A)** The total phenolic contents (TPC), **(B)** The results of DPPH (expressed in μmol TE/g), **(C)** DPPH radical scavenging activity, **(D)** The results of ABTS (expressed in μmol TE/g), **(E)** ABTS radical scavenging activity. Values are expressed as mean ± SD (*n* = 3). Bars with different lowercase letters represent significant difference (*P* < 0.05). Bars with ^*^ represent significant difference between USB and FSB under the same concentration (*P* < 0.05).

Microbial enzymes cause the free phenolic compounds to break free from their bound form (50). Phenolic compounds, which serve as metal chelators, hydrogen donors, reducing agents (free radical terminators), and singlet oxygen quenchers, constitute the majority of antioxidants. The antioxidative activity of food is highly associated with the total phenolic compounds (3, 51). Phenolic compounds found in soybean consist of phenolic acids (including ferulic acid, p-coumaric acid, vanillic acid, chlorogenic acid, caffeic acid, syringic acid, salicylic acid, protocatechuic acid, etc.), isoflavones (daidzein, glycitein, genistein and their glucosides) and anthocyanins (52). Phenolic compounds such as phenolic acids (gallic, caffeic, and p-coumaric acids, benzoic acid and cinnamic acid derivative), flavonoids (myricetin, rutin, naringenin, quercetin, morin, and hesperetin), coumarins, quinones, and styrylpyrones also exist in oyster mushrooms (53). Additionally, during fermentation, legumes produce more beneficial phenolic compounds because the microorganisms secreting ligninolytic and carbohydrate-metabolizing enzymes hydrolyze the phenolic glycosides to release free aglycones (51, 54). Therefore, it is assumed that the application of oyster mushroom (*P. ostreatus*) mycelium fermentation can greatly increase the content of phenolic compounds in soybean. In this study, the TPC of the unfermented soybeans was 4.62 mg/g. After SSF, the TPC of the fermented soybeans increased to 20.72 mg/g, marking a 4.47-fold increase compared to the control (*P* < 0.05) (Figure 1A). The fermentation of oyster mushroom mycelium led to a significant increase in total phenolic compounds, surpassing the rise observed in other fermented soybean products. For instance, when black beans were solid-state fermented using various Generally Recognized as Safe (GRAS) filamentous fungi, particularly *Aspergillus awamori*, the TPC of fermented soybeans increased from approximately 16 to 27.2 mg gallic acid equivalent/g (55). Similarly, employing *B. subtilis* BCRC 14715 during solid state fermentation resulted in an increase in the TPC of black soybean methanolic extract from 17.75 to 22.66 mg gallic acid equivalent/g (56). Furthermore, the TPC of Douchi was found to be 75.5% higher than that of unfermented soybean (57). TPC levels in commercially fermented soybean products from China with the highest TPC concentration being 12.29 ± 1.21 (mg GAE/g) were lower than the TPC level in this study (57). Nevertheless, Shukla et al. observed TPC values (22.2 30.6 mg GAE/g) in Korean fermented soybean paste, which were comparatively higher than the results of our analysis (58).

### 3.2 Assay of antioxidant activity of fermented soybeans by *P. ostreatus*

**Figure 2:**
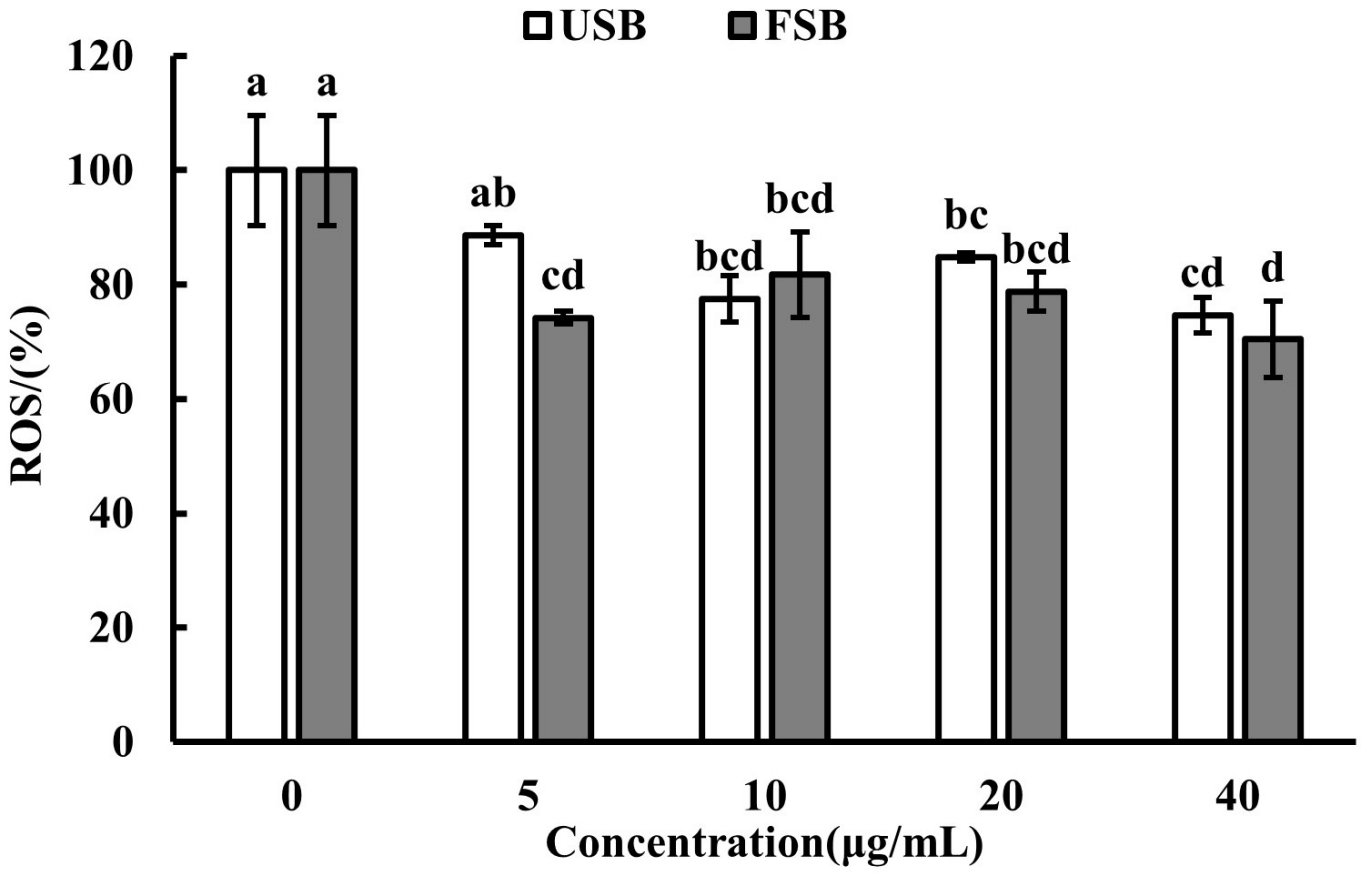
Reactive oxygen species (ROS) levels of LPS-induced RAW 264.7 macrophages treated by unfermented soybeans (USB) and fermented soybeans (FSB). Values are expressed as mean ± SD (*n* = 3). Bars with different lowercase letters represent significant difference (*P* < 0.05).

**Figure 3:**
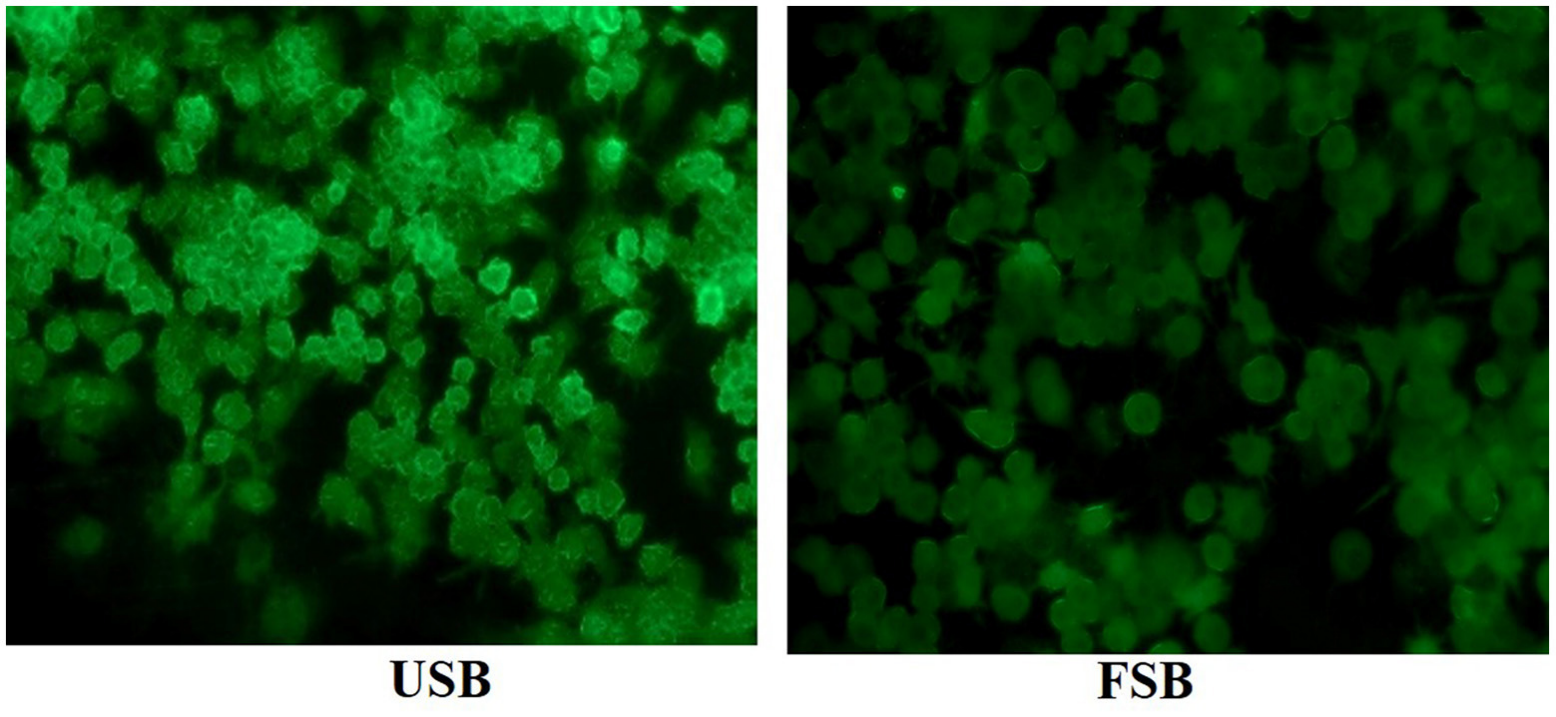
Fluorescence images of LPS-induced RAW 264.7 macrophages treated with methanol extracts (5 μg/mL) of unfermented soybeans (USB) and fermented soybeans (FSB).

Different antioxidant methods have been introduced to measure the antioxidant capacity of antioxidants in order to meet the assays for both hydrophilic and lipophilic antioxidants as well as the use of different radical sources. These methods can be separated into two categories: single electron transfer (SET) and hydrogen atom transfer (HAT) methods based on the reaction processes (59). The 1,1-Diphenyl-2-picrylhydrazyl (DPPH) radical scavenging assay is a widely utilized technique that provides an initial method for measuring antioxidant activity (60). It is now widely accepted that the direct HAT mechanism mediates the interaction between DPPH and phenols (59). The ABTS scavenging assay can be used with hydrophilic or lipophilic compounds. The reactions with ABTS radicals involve both HAT and SET mechanisms, in contrast to the HAT-only reactions with DPPH radicals (59, 61). The use of cell models to investigate a compound's antioxidant activity has been expanding in prominence since they allow for a more realistic biological environment (62). Using a combination of DPPH scavenging assay, ABTS scavenging assay, and cellular ROS assays in this study, the antioxidant activity of the fermented soybean methanolic extracts were assessed in comparison to the unfermented soybeans. The results are displayed in Figures 1–3. In terms of scavenging DPPH radicals, the fermented soybeans exhibited a value of 20.03 μmol TE/g. It was 3.92 times greater than that of the unfermented soybeans, which had a value of 5.11 μmol TE/g (Figure 1B). The DPPH scavenging activity of fermented soybeans increased gradually at concentrations of 0.5~4 mg/mL, and at each dose, the efficiency of scavenging radicals was higher than that of the unfermented soybeans (Figure 1C). The DPPH value in this study was similar to the highest values (20.58 ± 0.60 μmol TE/g) that have been documented for Chinese commercially fermented soybeans (57). Consistent with the finding of this study, DPPH radical scavenging activities of soybeans fermented by basidiomycetes (*Ganoderma lucidum, Hericium ramosum* and *Hericium erinaceus*) were significantly higher than those of unfermented soybeans (63). After fermentation using a single culture of *Rhizopus oligosporus*, the DPPH scavenging activity of soybeans increased dramatically, with antioxidants soluble in water and ethanol increasing by 4.3 and 3.7 times, respectively, compared to unfermented soybeans (64). In the ABTS assays, unfermented soybeans exhibited a scavenging activity of 15.98 μmol TE/g for ABTS radicals, while following SSF, the scavenging activity of fermented soybeans for ABTS radicals increased by 2.13-fold to 34.09 μmol TE/g (Figure 1D). The level of ABTs in the Douchi fermented soybean product with edible mushroom for 30 days, however, was less than the amount in this study, at around 28 μmol TE/g (65). The effectiveness of scavenging ABTS radicals was higher for fermented soybeans at each dose compared to unfermented soybeans, and the ABTS scavenging activity of the fermented soybeans increased progressively at concentrations of 2~8 mg/mL (Figure 1E). Soybeans fermented by *Cordyceps militaris* also demonstrated a significant scavenging effect against DPPH and ABTS radicals in a dose-dependent manner at each concentration level (66). Similarly, the scavenging activities of DPPH and ABTS increased in CKJ (*cheonggukjang*) (67). The elevated levels of DPPH and ABTS corroborated the findings of a prior study (65), which reported that after 30 days of fermentation, fermented soybeans exhibited a greater capacity for antioxidants than raw soybeans. Through metabolism and modification, phenolic chemicals become more bioavailable, which also helps to boost antioxidant activity during fermentation (3). It is widely acknowledged that significant antioxidant activities of food products are related to TPC (68). However, fungal fermentation is a complex metabolic process that can yield various antioxidant compounds, including phenolic compounds, antioxidant peptides and exopolysaccharides (EPS) (69). Furthermore, 80% methanol extracts may contain a variety of materials with antioxidant activity that function in different ways. The results further demonstrated that fermented soybeans possessed greater antioxidant capacities than unprocessed soybeans (57).

ROS are generated when macrophages are attacked by LPS. ROS play a role in controlling gene expression and activating cell signaling when macrophages participate in host cell defense mechanisms (70). In this study, intracellular ROS levels were measured using DCFH-DA, and the results are shown in Figure 2. The cell viability of RAW 264.7 macrophages did not significantly differ between the 0–40 μg/mL fermented soybeans under LPS induction (Data not shown). The ROS fluorescence intensity significantly decreased with the addition of 5 μg/mL fermented soybeans compared to unfermented soybeans indicating that fermented soybeans could inhibit the generation of ROS (Figure 2). Fluorescence images of fermented soybeans also confirmed this result (Figure 3). However, higher concentrations of unfermented soybean and fermented soybean extracts did not differ significantly in the production of ROS. This finding is consistent with previous reports (70), which found that black bean steamed liquid lyophilized product reduced ROS levels in LPS-induced RAW 264.7 macrophages. Therefore, fermented soybeans within a certain concentration can prevent macrophages from expressing ROS to mitigate intracellular oxidative stress.

Despite differing views among the scientific community, the methods used to evaluate antioxidant activity *in vitro* are inexpensive, simple to use, don't require extremely sensitive equipment, and aid in the planning of upcoming bioactive chemical studies (71). Considering the results of this study cannot be instantly applied to the “prevention, treatment, or cure” of non-communicable diseases in humans, the mechanisms of action behind the apparent antioxidant activity of fermented soybean *in vivo* must be elucidated.

### 3.3 Analysis of metabolite differences of fermented soybeans by *P. ostreatus*

**Figure 4:**
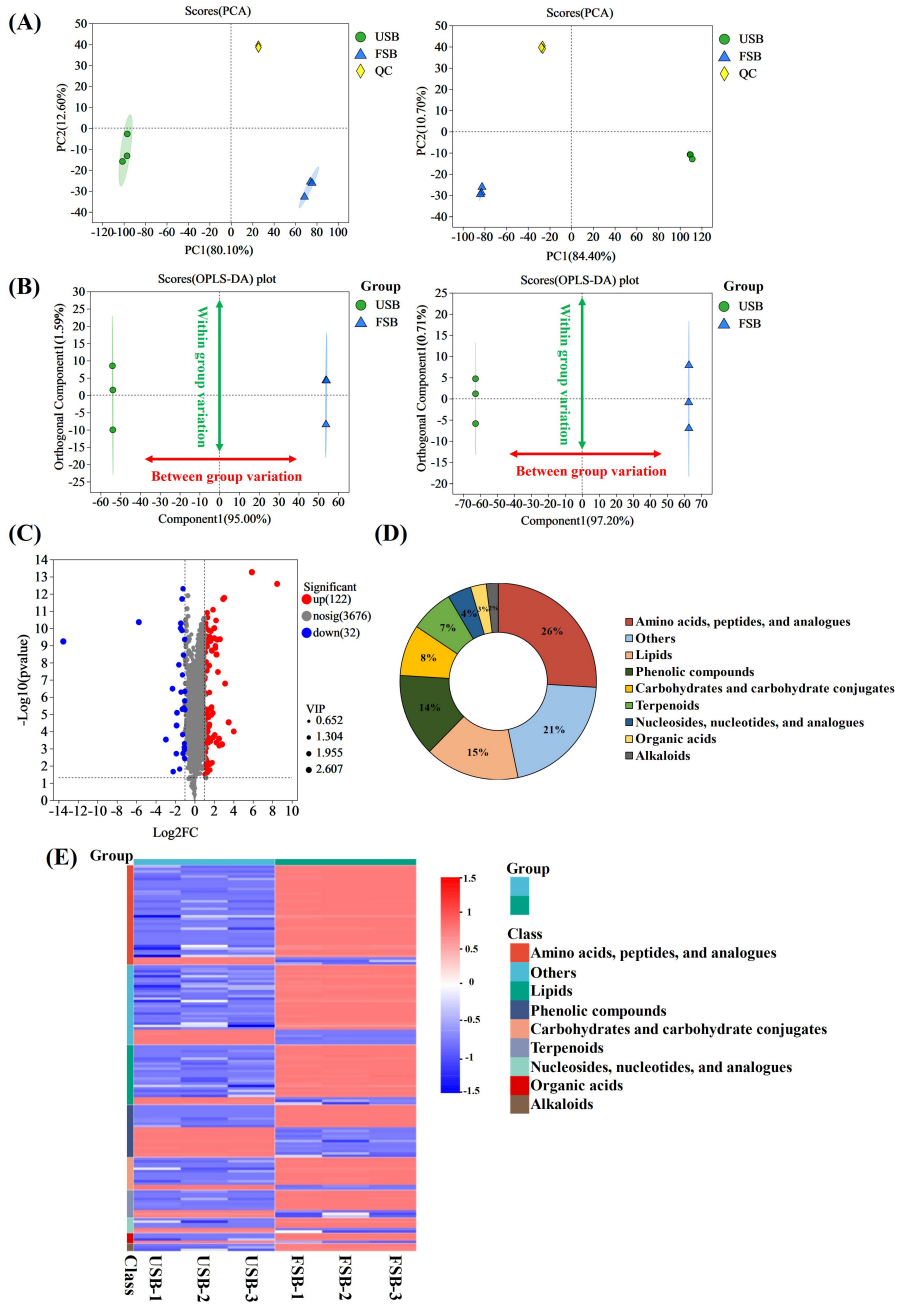
Analysis of different metabolites between unfermented soybeans (USB) and fermented soybeans (FSB). **(A)** Principal component analysis, **(B)** Orthogonal Partial Least Squares Discriminant Analysis (OPLS-DA), **(C)** Volcano plots of different metabolites, **(D)** Classification of different metabolites, **(E)** Clustering heatmaps of different metabolites were carried out to determine the differences in non-volatile metabolites between unfermented and fermented soybean samples. By VIP ≥ 1, FC ≥ 2 or ≤ 0.5 and *P* < 0.001 were used as screening conditions to identify differential metabolites.

The primary and secondary metabolites produced by microorganisms during fermentation change the properties of fermented foods. Therefore, monitoring small molecule metabolites in fermented foods using metabolomics is crucial for evaluating their functional properties (72). PCA, based on multivariate statistics, revealed the overall differences in metabolites of unfermented and fermented soybeans. The PCA scores of samples (including QC samples) are shown in Figure 4A. In the cationic mode, the first and second principal components explained 80.1% and 12.6% of the total variation, respectively. All QC samples were tightly clustered together, indicating good analytical stability and experimental repeatability (73). The results indicated a relative aggregation among the three parallel samples of the same treatment, with no significant difference, while two distinct clusters were observed in unfermented soybeans and fermented soybeans. This clear distinction between soybeans before and after fermentation suggests that their metabolism undergoes significant changes. This finding aligns with a previous study (74), indicating that fermentation has a significant effect on metabolites. The highlighted metabolites that contributed to the metabolic changes were further investigated using the OPLS-DA approach (Figure 4B).

OPLS-DA analyses were carried out to determine the differences in non-volatile metabolites between unfermented and fermented soybeans. The VIP (Variable Importance in Projection), fold change (FC) and *P*-value were utilized to screen for differential metabolites. The VIP value generated from the OPLS-DA model serves as a crucial indicator, filtering out the variables that contribute significantly to the model. It is generally believed that metabolites with a VIP value of ≥ 1 are deemed significantly different. FC represents the extent of difference in substance expression between two groups of samples; a FC of ≥ 2 indicates up-regulation, and a FC of ≤ 0.5 indicates down-regulation. A *P* < 0.001 indicates a significant difference. Therefore, metabolites with VIP values ≥ 1, FC values ≥ 2 or ≤ 0.5, and *P* < 0.001 were considered significantly different (75). Previous research suggests that carbohydrates, amino acids, organic acids, nucleotides, lipids, flavonoids, phenolic acids, alkaloids, terpenoids, tannins, coumarins and lignin are among the main differential metabolites following SSF (65, 74, 76). In this study, a total of 154 different metabolites were identified after fermentation, consisting of 122 up-regulated metabolites, and 32 down-regulated metabolites (Figure 4C). These metabolites included 40 amino acids, peptides, and analogs; 32 others; 24 lipids; 21 phenolic compounds; 13 carbohydrates and carbohydrate conjugates; 11 terpenoids; 6 nucleosides, nucleotides, and analogs; 4 organic acids and 3 alkaloids (Figure 4D). After SSF, amino acids, carbohydrates and their derivatives, and phenolic compounds emerged as the main differential metabolites, which are related to the whole complex growth metabolism during fungal fermentation, including the synthesis of amino acids, the release of active peptides, the degradation of cellulose, the hydrolysis of bound phenols, and the generation of new active ingredients (77). *P. ostreatus* may grow in almost any organic substrate generated by our food production system (78), So *P. ostreatus* also grew in soybean and degraded protein, carbohydrate, lipid, and other components of soybean into small molecular compounds including amino acids, peptides, carbohydrates and free fatty acids to survive. On the other hand, *P. ostreatus* is an outstanding microbial cell manufacturer that produces secondary metabolites. The mycelium of six species of the genus Pleurotus were discovered to contain a wide variety of secondary metabolites including phenolic compounds, cinnamic acid, phenylalanine, indole derivatives, sterols, terpenoids, lovastatin, and ergothioneine (79, 80). To better understand the non-volatile metabolites of soybeans during fermentation, a heatmap (Figure 4E) was employed as the basis for hierarchical cluster analysis (HCA). Consistent with the PCA results, the results indicated that the three biological replicates were clustered together because of the relative contents of non-volatile metabolites.

Notably, we discovered that N-Jasmonoylisoleucine (JA-Ile) was 2.49 times more abundant in fermented soybeans than in unfermented soybeans (Supplementary Table S1). The jasmonate signal molecule JA-Ile is generated from polyunsaturated fatty acids (81), and plays primary roles in regulating numerous ecological interactions with biotic and abiotic environments. JA-Ile could induce the biosynthesis of diverse secondary metabolites such as terpenoids, phenylpropanoids, phenols and alkaloids (82, 83). It was reported that the defense hormone jasmonate regulation in *P. ostreatus* triggered the mycelial production of terpenoids and steroids in response to fungivore attacks (84). In this study, JA-Ile-mediated core signaling may also play a key role in regulating the biosynthesis of a wide variety of secondary metabolites by solid-state fermentation of soybeans using *P. ostreatus*.

### 3.4 Analysis of different metabolites and correlation with antioxidant capacities

**Figure 5:**
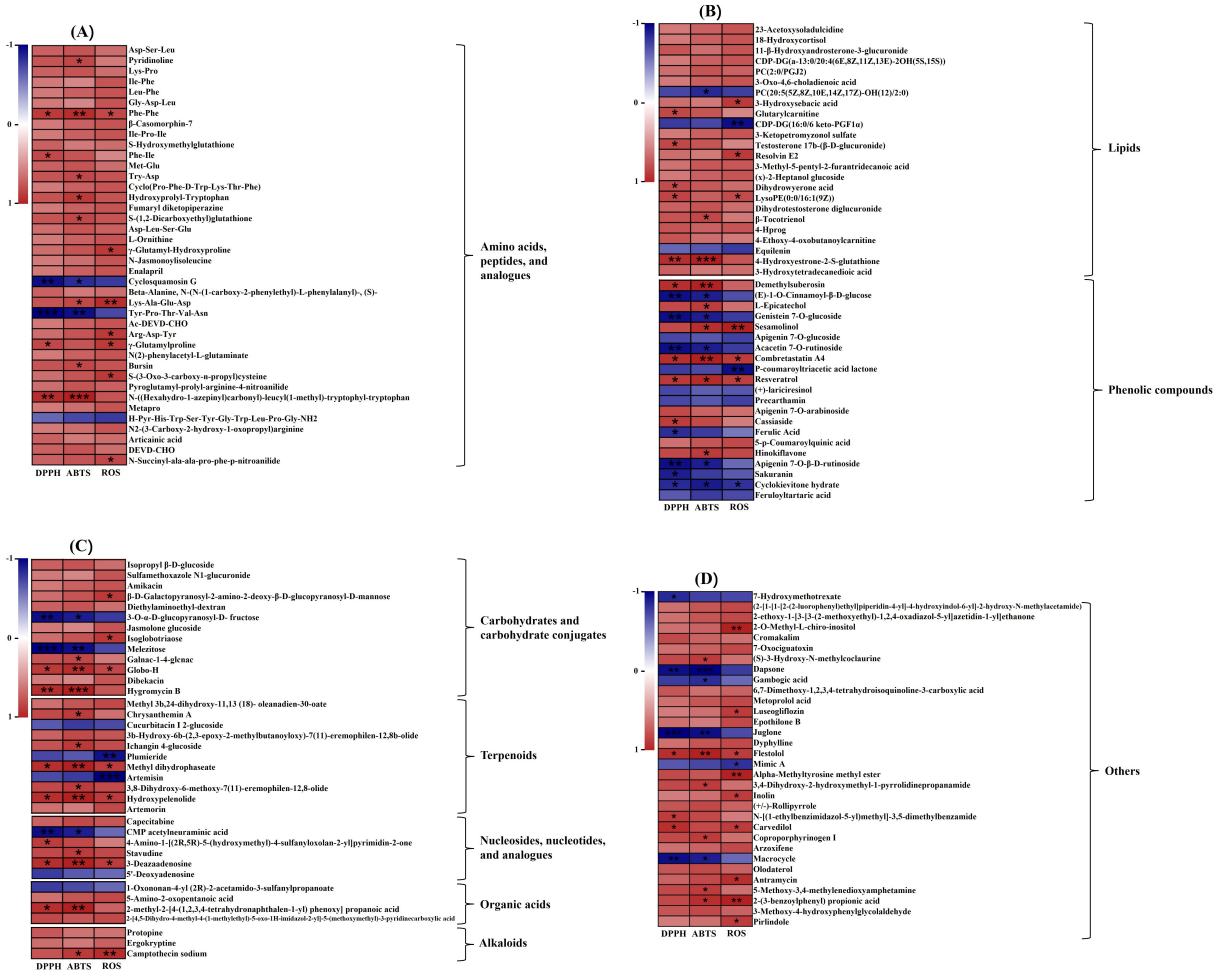
The heatmaps of the correlation analysis between 154 differential metabolites and antioxidant activity. **(A)** Amino acids, peptides, and analogs, **(B)** lipids and phenolic compounds, **(C)** carbohydrates and carbohydrate conjugates, terpenoids, nucleosides, nucleotides, and analogs, organic acids and alkaloids, **(D)** others (*P < 0.05; **P < 0.01; ***P < 0.001).

The Correlation between metabolites and antioxidant capacity are presented in Figure 5. As shown in Figure 5, 58 metabolites showed positive correlations with the antioxidant activity of the fermented soybean, while 23 metabolites had negative associations.

#### 3.4.1 Amino acids, peptides, and analogs

In the OPLS-DA analysis comparing fermented and unfermented soybeans, 40 differential metabolites of amino acids, peptides, and analogs were identified. Among them, amino acids, peptides and analogs are the most varied metabolites (Supplementary Table S1). During soybean fermentation, several oligopeptides were released through proteolytic processes involving the breakdown by proteolytic enzymes generated from *P. ostreatus* mycelium. It had been reported that the hydrophobic amino acid especially phenylalanine (Phe) was exposed to hydroxyl radicals and converted to tyrosine to scavenge hydroxyl radicals or reduce metal cations (85). Higher antioxidant activity was also attributed to the aromatic amino acids tyrosine (Tyr), tryptophan (Trp), cysteine (Cys) and methionine (Met), which could supply electrons or hydrogen atoms (86). The hydrophobic amino acids may help reduce peroxidation by making peptides more soluble in lipids and improving their ability to interact with radical species (87). Tyr is produced when Phe is exposed to hydroxyl radicals, and Tyr exhibits the hydroxyl radical's scavenging ability (88). Pro increased antioxidant enzyme activities such as ascorbate peroxidase and peroxidase (89). Pro-based peptides are widely recognized for their essential roles in signal transduction pathways and their antibacterial, immunomodulatory, and antioxidant characteristics, among other health-promoting activities (90, 91). Correlation analysis thus revealed the increased antioxidant activities of dipeptides and other oligopeptides that comprised hydrophobic amino acids, aromatic amino acids, and Pro (Figure 5A). Dipeptides could be fully absorbed from the intestinal lumen into the bloodstream, which resulted in the expression of biological actions at the tissue levels (92). Dipeptides through fermentation exhibited antioxidant activity and angiotensin I-converting enzyme suppressive behavior (93, 94). Dipeptides associated with antioxidant activity including phenylalanine-phenylalanine (Phe-Phe), phenylalanine-isoleucine (Phe-Ile), tryptophan-aspartate (Try-Asp), hydroxyprolyl-tryptophan, γ-Glutamylhydroxyproline and γ-Glutamylproline were among the metabolites contributing mainly to the observed discrimination in this study (Figure 5A). The amounts of dipeptides containing Phe, Phe-Phe, and Phe-Ile in the fermented group increased significantly (*P* < 0.001) in comparison to the unfermented group, reaching values 6.09 times and 2.86 times higher, respectively (Table 2). Additionally, the results of the correlation assay indicated that the other oligopeptides containing hydrophobic and aromatic amino acids might have antioxidant qualities (Figure 5A). On the contrary, the contents of cyclosquamosin G and Tyr-Pro-Thr-Val-Asn declined after fermentation, and there was negative correlation between their antioxidant activity and content (Table 2, Figure 5A). The results made in this study require additional verification as there are currently no studies on the relationship between these two chemicals and antioxidant activities.

**Table 2:** 58 differential metabolites related to antioxidant activity of fermented soybeans.

**Category**	**Metabolite**	**VIP**	***P* value**	**Abundance**		**FC**	**M/Z**
				**USB** ^a^	**FSB** ^b^		
Amino acids, peptides, and analogs	Pyridinoline	2.36	2.01E-12	0.82 ± 0.01	6.28 ± 0	7.62	427.18
	Phe-Phe	2.25	4.32E-10	0.98 ± 0.02	5.95 ± 0.01	6.09	311.14
	Phe-Ile	2.15	3.94E-05	2.17 ± 0.35	6.2 ± 0.01	2.86	279.17
	Try-Asp	2.11	8.37E-12	1.61 ± 0.01	5.96 ± 0	3.71	318.11
	Hydroxyprolyl-Tryptophan	2.08	6.76E-04	0.92 ± 0.79	5.27 ± 0.02	5.75	298.12
	S-(1,2-Dicarboxyethyl) glutathione	2.03	9.48E-11	3.22 ± 0.01	7.27 ± 0.01	2.26	422.09
	γ-Glutamyl-Hydroxyproline	2.02	1.23E-09	2.65 ± 0.01	6.21 ± 0.02	2.35	584.22
	Cyclosquamosin G	2.01	3.61E-09	6.4 ± 0.02	2.88 ± 0.02	0.45	846.42
	Lys-Ala-Glu-Asp	1.99	6.57E-07	3.09 ± 0.12	6.97 ± 0	2.25	460.21
	Tyr-Pro-Thr-Val-Asn	1.97	1.17E-03	6.72 ± 0.03	3.23 ± 0.73	0.48	593.29
	Arg-Asp-Tyr	1.95	3.56E-03	2.97 ± 0.98	6.45 ± 0.03	2.17	453.21
	γ-Glutamylproline	1.94	4.67E-06	3.65 ± 0.19	7.36 ± 0	2.02	243.10
	Bursin	1.89	6.34E-11	2.97 ± 0.01	6.47 ± 0.01	2.18	374.17
	S-(3-Oxo-3-carboxy-n-propyl) cysteine	1.88	2.92E-08	2.98 ± 0.05	6.46 ± 0.01	2.17	220.03
	N-((Hexahydro-1-azepinyl)carbonyl)-leucyl(1-methyl)-tryptophyl-tryptophan	1.87	7.60E-10	2.71 ± 0.01	6.15 ± 0.02	2.27	656.29
	N-Succinyl-ala-ala-pro-phe-p-nitroanilide	1.75	3.10E-02	2.91 ± 1.66	6.03 ± 0.01	2.07	642.29
Lipids	PC[20:5(5Z,8Z,10E,14Z,17Z)-OH(12)/2:0]	2.04	9.93E-11	6.44 ± 0.01	2.35 ± 0	0.37	644.33
	3-Hydroxysebacic acid	2.04	6.46E-05	3.12 ± 0.36	6.76 ± 0.02	2.17	260.15
	Glutarylcarnitine	2.01	1.01E-04	2.26 ± 0.39	5.78 ± 0.01	2.56	276.14
	CDP-DG(16:0/6 keto-PGF1α)	1.97	8.32E-04	6.6 ± 0.05	3.14 ± 0.66	0.48	534.76
	Testosterone 17b-(β-D-glucuronide)	1.95	6.83E-05	2.44 ± 0.38	6.17 ± 0.01	2.53	499.21
	Resolvin E2	1.93	5.11E-06	1.91 ± 0.17	5.16 ± 0.04	2.70	357.20
	Dihydrowyerone acid	1.86	1.62E-04	2.94 ± 0.43	6.35 ± 0.01	2.16	291.09
	LysoPE(0:0/16:1(9Z))	1.85	3.59E-04	2.17 ± 0.52	5.57 ± 0.02	2.57	472.24
	β-Tocotrienol	1.82	1.42E-02	2.31 ± 1.34	5.51 ± 0.05	2.39	393.31
	4-Hydroxyestrone-2-S-glutathione	1.75	9.58E-10	2.94 ± 0.01	5.95 ± 0.02	2.02	590.21
Phenolic compounds	Demethylsuberosin	2.61	5.48E-14	0.11 ± 0	6.78 ± 0	59.13	267.04
	(E)-1-O-Cinnamoyl-β-D-glucose	2.58	5.83E-10	5.79 ± 0.03	0 ± 0	0.00	621.22
	L-Epicatechol	2.36	2.62E-13	0.02 ± 0	5.5 ± 0	358.11	289.07
	Genistein 7-O-glucoside	2.34	4.52E-05	6.61 ± 0.04	1.82 ± 0.44	0.28	433.11
	Sesamolinol	2.22	3.75E-10	1.42 ± 0.01	5.72 ± 0.02	4.02	414.15
	Acacetin 7-O-rutinoside	2.17	5.09E-06	7.02 ± 0.02	2.93 ± 0.21	0.42	606.19
	Combretastatin A4	2.16	1.41E-09	1.37 ± 0.01	5.96 ± 0.03	4.36	677.26
	P-coumaroyltriacetic acid lactone	2.16	3.29E-07	5.76 ± 0.02	1.18 ± 0.12	0.21	252.04
	Resveratrol	2.12	3.52E-11	1.24 ± 0.01	5.66 ± 0.01	4.58	273.08
	Cassiaside	2.01	5.13E-06	1.81 ± 0.21	5.8 ± 0.01	3.21	385.09
	Ferulic acid	1.96	1.68E-06	6.53 ± 0.01	3.19 ± 0.13	0.49	177.05
	Hinokiflavone	1.90	4.33E-04	3.02 ± 0.58	6.61 ± 0.02	2.19	537.08
	Apigenin 7-O-β-D-rutinoside	1.85	5.18E-08	5.8 ± 0.06	2.43 ± 0	0.42	577.16
	Sakuranin	1.82	5.18E-07	5.2 ± 0.03	1.96 ± 0.09	0.38	447.13
	Cyclokievitone hydrate	1.77	5.05E-04	6.02 ± 0.04	2.89 ± 0.53	0.48	803.26
Carbohydrates and carbohydrate conjugates	β-D-Galactopyranosyl-2-amino-2-deoxy-β-D-glucopyranosyl-D-mannose	2.28	4.64E-10	2.28 ± 0.01	6.78 ± 0.02	2.97	545.22
	3-O-α-D-glucopyranosyl-D- fructose	2.05	5.45E-06	7.3 ± 0.01	3.64 ± 0.2	0.50	365.11
	Isoglobotriaose	1.96	2.48E-09	2.9 ± 0.01	6.23 ± 0.02	2.15	520.23
	Melezitose	1.96	4.48E-10	6.48 ± 0.02	3.16 ± 0.01	0.49	527.16
	Galnac-1-4-glcnac	1.92	5.04E-11	3.04 ± 0.01	6.68 ± 0.01	2.19	405.15
	Globo-H	1.82	9.39E-09	3.18 ± 0.01	6.42 ± 0.03	2.02	574.20
	Hygromycin B	1.80	7.02E-10	3.02 ± 0.01	6.19 ± 0.02	2.05	572.23
Terpenoids	Chrysanthemin A	2.35	1.71E-12	0.75 ± 0.01	6.15 ± 0	8.24	615.25
	Ichangin 4-glucoside	2.08	4.56E-04	2.3 ± 0.63	6.12 ± 0.04	2.66	692.29
	Plumieride	2.05	2.19E-02	6.01 ± 0.02	1.3 ± 2.24	0.22	451.12
	Methyl dihydrophaseate	2.00	3.08E-10	2.04 ± 0.01	5.95 ± 0.02	2.92	317.14
	Artemisin	1.95	1.92E-03	6.09 ± 0.04	2.66 ± 0.82	0.44	301.09
	3,8-Dihydroxy-6-methoxy-7(11)-eremophilen-12,8-olide	1.93	5.40E-07	1.95 ± 0.11	5.59 ± 0.01	2.87	317.14
	Hydroxypelenolide	1.86	2.69E-08	2.42 ± 0.05	5.81 ± 0	2.40	289.12
Nucleosides, nucleotides, and analogs	Cmp-nana	2.13	1.97E-12	7.5 ± 0	3.06 ± 0	0.41	595.13
	4-Amino-1-[(2R,5R)-5-(hydroxymethyl)-4-sulfanyloxolan-2-yl]pyrimidin-2-one	2.01	8.16E-03	2.04 ± 1.35	5.83 ± 0.01	2.87	276.10
	Stavudine	1.97	1.24E-11	2.63 ± 0.01	6.44 ± 0	2.45	447.15
	3-Deazaadenosine	1.91	8.96E-09	2.78 ± 0.04	6.35 ± 0.01	2.28	301.07
Organic acids	2-methyl-2-[4-(1,2,3,4-tetrahydronaphthalen-1-yl) phenoxy] propanoic acid	1.96	2.37E-11	2.5 ± 0.01	6.29 ± 0.01	2.51	679.33
Alkaloids	Camptothecin sodium	2.01	6.63E-03	1.72 ± 1.42	5.95 ± 0.01	3.47	401.09
Others	7-Hydroxymethotrexate	2.38	4.41E-11	5.65 ± 0.02	0.11 ± 0	0.02	491.14
	2-O-Methyl-L-chiro-inositol	2.17	4.49E-04	1.26 ± 0.77	5.98 ± 0.01	4.73	387.15
	(S)-3-Hydroxy-N-methylcoclaurine	2.03	1.49E-10	1.87 ± 0.01	5.93 ± 0.01	3.17	336.12
	Dapsone	2.03	1.34E-08	5.29 ± 0.04	1.71 ± 0.02	0.32	519.11
	Gambogic acid	2.02	4.38E-06	6.57 ± 0.02	3.02 ± 0.18	0.46	646.34
	Luseogliflozin	2.00	8.17E-03	3.01 ± 1.33	6.76 ± 0.02	2.24	467.21
	Juglone	1.99	4.75E-07	6.76 ± 0.01	3.33 ± 0.1	0.49	207.07
	Flestolol	1.94	5.32E-06	2.81 ± 0.2	6.52 ± 0.01	2.32	372.16
	Mimic A	1.93	5.02E-13	6.46 ± 0	2.81 ± 0	0.44	565.21
	Alpha-Methyltyrosine methyl ester	1.93	2.75E-04	2.57 ± 0.53	6.26 ± 0.01	2.43	254.10
	3,4-Dihydroxy-2-hydroxymethyl-1-pyrrolidinepropanamide	1.92	1.62E-03	2.84 ± 0.76	6.16 ± 0.02	2.17	431.21
	Inolin	1.91	1.75E-02	2.05 ± 1.78	6.06 ± 0.01	2.96	366.13
	N-[(1-ethylbenzimidazol-5-yl)methyl]-3,5-dimethylbenzamide	1.89	7.92E-05	2.66 ± 0.33	5.79 ± 0.03	2.18	308.18
	Carvedilol	1.88	8.05E-09	3.44 ± 0.03	6.9 ± 0.01	2.00	451.18
	Coproporphyrinogen I	1.85	9.21E-10	2.7 ± 0.01	6.04 ± 0.02	2.24	705.31
	Macrocycle	1.78	4.37E-10	6.18 ± 0.02	3.07 ± 0	0.50	551.15
	Antramycin	1.72	8.74E-06	2.9 ± 0.18	5.83 ± 0	2.01	360.12
	5-Methoxy-3,4-methylenedioxyamphetamine	1.71	9.90E-10	2.07 ± 0.01	4.95 ± 0.02	2.39	254.10
	2-(3-benzoylphenyl) propionic acid	1.69	7.39E-03	1.94 ± 1.05	4.97 ± 0.02	2.56	299.09
	Pirlindole	1.62	6.27E-06	2.52 ± 0.14	5.09 ± 0.02	2.02	271.15

^a^USB represents unfermented soybeans.

^b^FSB represents fermented soybeans.

#### 3.4.2 Lipids

Lipids are essential components for cells and have been demonstrated to play a vital role in foods undergoing microbial fermentation (95). In this study, the lipid content in the fermented soybeans increased from 19.38% to 20.34% (Table 1), indicating the generation of more lipids during SSF. The variations in phospholipids during fermentation could lead to modifications in the structure of cellular membranes, influencing various biological functions associated with phospholipids (96). In this study, the levels of CDP-DG[a-13:0/20:4(6E,8Z,11Z,13E)-2OH(5S,15S)] and PC(2:0/PGJ2) significantly increased after soybean fermentation compared to the unfermented soybeans, while the levels of CDP-DG(16:0/6 keto-PGF1α) and PC[20:5(5Z,8Z,10E,14Z,17Z)-OH(12)/2:0] decreased (*P* < 0.001). Enzymes are known to hydrolyze phospholipids into LPC, LPE and fatty acids (65, 75). In addition, the content of Lyso PE [0:0/16:1(9Z)] increased significantly, reaching a 2.57- fold increase compared to unfermented soybeans after fermentation (Supplementary Table S1). According to the results, fermentation significantly impacted soybean free fatty acid such as 3-Hydroxysebacic acid, 3-Methyl-5-pentyl-2-furantridecanoic acid and dihydrowyerone acid (*P* < 0.001) (Table 2). Correlation analysis showed that the contents of these three fatty acids were positively correlated with antioxidant activity (Figure 5B). The unsaturated fatty acids showed a high sensitivity to oxidation and were easily peroxidized by OH-attack. Polyunsaturated fatty acid peroxidation produces isoprostanes (97), and their concentrations are thought to be a good indicator of oxidative stress (98). It remains to be confirmed whether the three fatty acids found in this study have the aforementioned effects. Rong et al. (65) discovered a relationship between free fatty acids and douchi's antioxidant activity, which is in agreement with the results of this investigation.

Notably, upregulated sterol derivatives contained 18-Hydroxycortisol, 11-β-Hydroxyandrosterone-3-glucuronide, 3-Oxo-4,6-choladienoic acid, 3-Ketopetromyzonol sulfate and testosterone 17b-(β-D-glucuronide), dihydrotestosterone diglucuronide and 23-Acetoxysoladulcidine in the metabolites of lipids (Supplementary Table S1). Ergosterol abundant in oyster mushrooms (99), may serve as the precursor for the synthesis of these sterol derivatives during soybean fermentation by *P. ostreatus* mycelium. This could potentially explain the high lipid content observed in fermented soybeans. Among these sterol derivatives, testosterone 17b-(β-D-glucuronide) was thought to be associated with antioxidant activity of fermented soybean in this study (Figure 5B). Thus far, there has been no related research showing a connection between this compound and antioxidant activity; more experiments are necessary to validate this result. In this study, the results of correlation analysis showed that β-Tocotrienol and 4-Hydroxyestrone-2-S-glutathione were positively correlated with antioxidant activity in the fermented soybean (Figure 5B). Vitamins E and glutathione were considered that the powerful antioxidants for their ability to combat reactive oxygen and nitrogen species (100). β-Tocotrienol and 4-Hydroxyestrone-2-S-glutathione are the derivatives of vitamins E and glutathione, which could explain the antioxidant activity of these two compounds.

#### 3.4.3 Phenolic compounds

The phenolic compounds including phenolic acids, coumarins, lignans, chalcones, avonoids, lignins, and stilbenes are the secondary metabolism of most fungus and plants, which are important phytochemicals with a broad range of antioxidant capacities (101). By supplying hydrogen atoms and generating antioxidant radicals, pheonlic compounds could react with free radicals and transform them into more stable products. These products were stabilized by the delocalization of the unpaired electron along the phenolic ring, which resulted in stable resonance hybrids (102). The majority of phenolic compounds found in unfermented soybeans are anthocyanins, phenolic acids (ferulic acid, *p*-coumaric acid, chlorogenic acid, caffeic acid, syringic acid, vanillic acid, salicylic acid, protocatechuic acid, etc.), and isoflavones (daidzein, glycitein, genistein, and their glucosides) (52). However, in this study, it was observed that coumarin, flavonoids, lignan and stilbene were the phenolic compounds that underwent the most significant changed during the solid-state fermentation of soybeans by *P. ostreatus* mycelium, rather than phenolic acids (Supplementary Table S1). A significant positive correlation was observed between antioxidant activity and the phenolic compounds including demethylsuberosin, L-Epicatechol, sesamolinol, combretastatin A4, resveratrol, hinokiflavone and cassiasside (Figure 5B). Coumarins exhibit a wide range of functions as antibacterial, anticancer, and anticoagulant agents (103). Demethylsuberosin, a coumarin, was found in fermented soybeans that had undergone fermentation at a level 59.12 times higher (Table 2). The presence of demethylsuberosin in *Angelica decursiva* and *Citrus aurantium* L. var. *amara* Engl was reported to be related to the ability to suppress lipid accumulation and lower blood pressure (104, 105). L-Epicatechol is the flavonoid compound with the largest FC among all the differential metabolites, and its content is 358.1 times that of unfermented soybeans. L-Epicatechol identified as a potent antioxidant compound in wild soybeans, with levels 1,750 times higher than those in cultivated soybeans (76). Sesamolinol, a lignan, significantly increased in concentration during fermentation, reaching levels 4.02 times higher than in unfermented soybeans. Sesamolinol has been reported to reduce oxidative stress and affect lipid metabolism in liver cells (106). In this study, the contents of combretastatin A4 and resveratrol in fermented soybeans were 4.36 times and 4.58 times higher than unfermented soybeans, respectively (Table 2). Combretastatin A4 and resveratrol are examples of stilbene substances. A powerful inhibitor of tubulin polymerization, combretastatin A4 has a significant suppressive effect on the development of tumor cells (107). Resveratrol possesses numerous biological properties and health benefits, including cardioprotective, neuroprotective, anti-inflammatory, anti-cancer, anti-diabetic, and antioxidant qualities (108, 109). Cassiaside is a phenolic glycoside analogous, and it was discovered that cassiaside exhibited hepatoprotective benefits and that the reduction in ROS generation in tert-butyl hydroperoxide induced HepG2 cells (110).

In the OPLS-DA analysis of the entire fermentation process, nine other flavonoids were among the differential phenolic compounds, including genistein 7-O-glucoside, apigenin 7-O-glucoside, acacetin 7-O-rutinoside, apigenin 7-O-arabinoside, hinokiflavone, apigenin 7-O-β-D-rutinoside, sakuranin, cyclokievitone hydrate and precarthamin (Supplementary Table S1). Among them, the contents of six flavonoid o-glycosides including genistein 7-O-glucoside, apigenin 7-O-glucoside, acacetin 7-O-rutinoside, apigenin 7-O-β-D-rutinoside, sakuranin and precarthamin, cyclokievitone hydrate (isoflavonoid) were significantly reduced, consistent with a previous study showing a decrease in flavonoid glycosides dropped after soybean fermentation (65). During fermentation, the avonoid glucosides including genistein 7-O-glucoside, acacetin 7-O-rutinoside, apigenin 7-O-β-D-rutinoside, sakuranin and cyclokievitone hydrate were negatively correlated with antioxidant activity, respectively (Figure 5B). Fermentation could facilitate the conversion of glycoside molecules, thereby enhancing the antioxidant capacity of soybeans (65). In addition, the health-promoting biflavone hinokiflavone (111) was found to be significantly elevated after SSF, increasing 2.19 times more than those in unfermented soybeans, and correlation analysis revealed a positive association between this component and the fermented soybean's antioxidant properties (Figure 5B).

#### 3.4.4 Carbohydrates and carbohydrate conjugates

The genus *Pleurotus* is among the most exploitable xylotrophic fungi, capable of producing ligninolytic enzymes such as MnP and laccase, in addition to complex enzymes such as endo-glucanase, exo-glucanase, β-glucosidase, endo-xylanase, xylosidase, endo-mannanase and mannosidase (112). The carbohydrates in fermented soybean include starch, cellulose, hemicellulose, and pectin. Under the action of the carbohydrate–active enzymes, the amounts of starch and dietary fiber decrease, while most monosaccharides and oligosaccharides increased gradually during SSF (113). As fermentation progresses, these sugars transformed into organic acids, sugar alcohols, or other sugar conjugates that participate in various metabolic processes (113, 114). The carbohydrate contents of fermented soybeans, particularly the concentration of monosaccharides or oligosaccharides, have shown conflicting findings due to the use of different fermentation strains, techniques, and durations (74, 114, 115). In this study, 13 differential metabolites of carbohydrates and carbohydrate conjugates were identified by the OPLS-DA analysis, and the metabolites were reported in Supplementary Table S1. Correlation heatmaps were generated to 7 potential carbohydrates and carbohydrate conjugates associations with the antioxidant activity of the soybeans fermented by *P. ostreatus* mycelium (Figure 5C). It was reported that oligosaccharides had the potential to enhance the activity of key antioxidant enzymes that scavenge reactive oxygen species (ROS) including peroxidase (POD), catalase (CAT) and superoxide dismutase (SOD) (116).

#### 3.4.5 Terpenoids

In this study, it was observed that sesquiterpenoids, triterpenoids and terpenoid glycosides changed most significantly in soybean after SSF. 7 sesquiterpenoids compounds were identified, including chrysanthemin A, 3b-Hydroxy-6b-(2,3-epoxy-2-methylbutanoyloxy)-7(11)- eremophilen- 12,8b-olide, methyl dihydrophaseate, artemisin, 3,8-Dihydroxy-6-methoxy-7(11)-eremophilen−12,8-olide, hydroxypelenolide and artemorin. Except for artemisin, the concentration of all sesquiterpenoids increased after SSF in this study (Supplementary Table S1). It was reported that sesquiterpenes constitute approximately 58% of the newly identified bioactive terpenes from endophytic fungi in the past decade, demonstrating various activities such as anti-viral, an-ti-microbial, antioxidant, anti-cancer, anti-acetylcholinesterase (AChE) properties (117). Our findings support this information. Sesterterpene synthase catalyzes the initial scaffold-generating step, which is primarily responsible for the structural variety of sesterterpenes. The sesterterpene synthases that have been found in fungi are bifunctional, having two domains: the terpene synthase domain and the trans-prenyltransferase domain, thus it is conducive to synthesize the product in a head-to-tail manner (80, 118). Among the triterpenoids, the level of methyl 3b,24-dihydroxy-11,13(18)-oleanadien−30-oate significantly increased, while the content of cucurbitacin I 2-glucoside decreased significantly after soybean fermentation. Triterpenoids, primarily soyasaponins, were found to be significant components of terpenoid differential metabolites in the soybean fermentation process (65). By eliminating the extra energy in singlet oxygen and releasing it as heat, the terpenoids serve as singlet oxygen quenchers, bringing the oxygen back to its unexcited state and allowing the terpenoids to be recycled as antioxidants (102). The results showed that antioxidant activity and five distinct terpenoids correlated positively, while antioxidant activity and two terpenoids correlated negatively (Figure 5C). There have been no reports of these terpenoids having any connection to antioxidant activity thus far. Fermentation might enhance the potential antioxidant activity and health benefits of soybean by up-regulation of terpene-active compounds.

#### 3.4.6 Other compounds

From the results of Figure 5D, it could be seen that other non-volatile metabolites also had a high contribution to antioxidant activity, such as alkaloids, organic acids, nucleosides, ketones, and aldehydes (119–122). Camptothecin as an indole alkaloid, a modified monoterpene found in some plants (angiosperms) and endophytic fungi, is the source of the major anticancer medicine (123). This study is the first to demonstrate camptothecin can also be created by SSF of soybean with *P. ostreatus* and have a potential antioxidant activity (Figure 5D). Juglone was negatively correlated with antioxidant activity in this study (Figure 5D). It was reported that juglone (5-hydroxyl-1,4-naphthoquinone) showed the antioxidant capacities, which could serve to combat oxidative stress (124). Due to the function of the ligninolytic enzymes from *P. ostreatus*, Juglone was downregulated after SSF (Table 2). Therefore, it was negatively correlated with antioxidant activity (Figure 5D). The methylated inositol 2-O-Methyl-L-chiro-inositol exhibited anti-diabetic, anti-platelet aggregation, and free radical scavenging activities (125). In this study's correlation analysis, we also discovered 2-O-Methyl-L-chiro-inositol's possible antioxidant properties (Figure 5D). Carvedilol was positively correlated with antioxidant activity in this study (Figure 5D). Though carvedilol is a non-selective β-blocker that is frequently used to treat ischemic heart disease and hypertension, it demonstrated strong antioxidant properties unrelated to its adrenergic receptor-blocking ability (126).

#### 3.4.7 Integration of metabolic pathway of the metabolites associated with antioxidant activity

**Figure 6:**
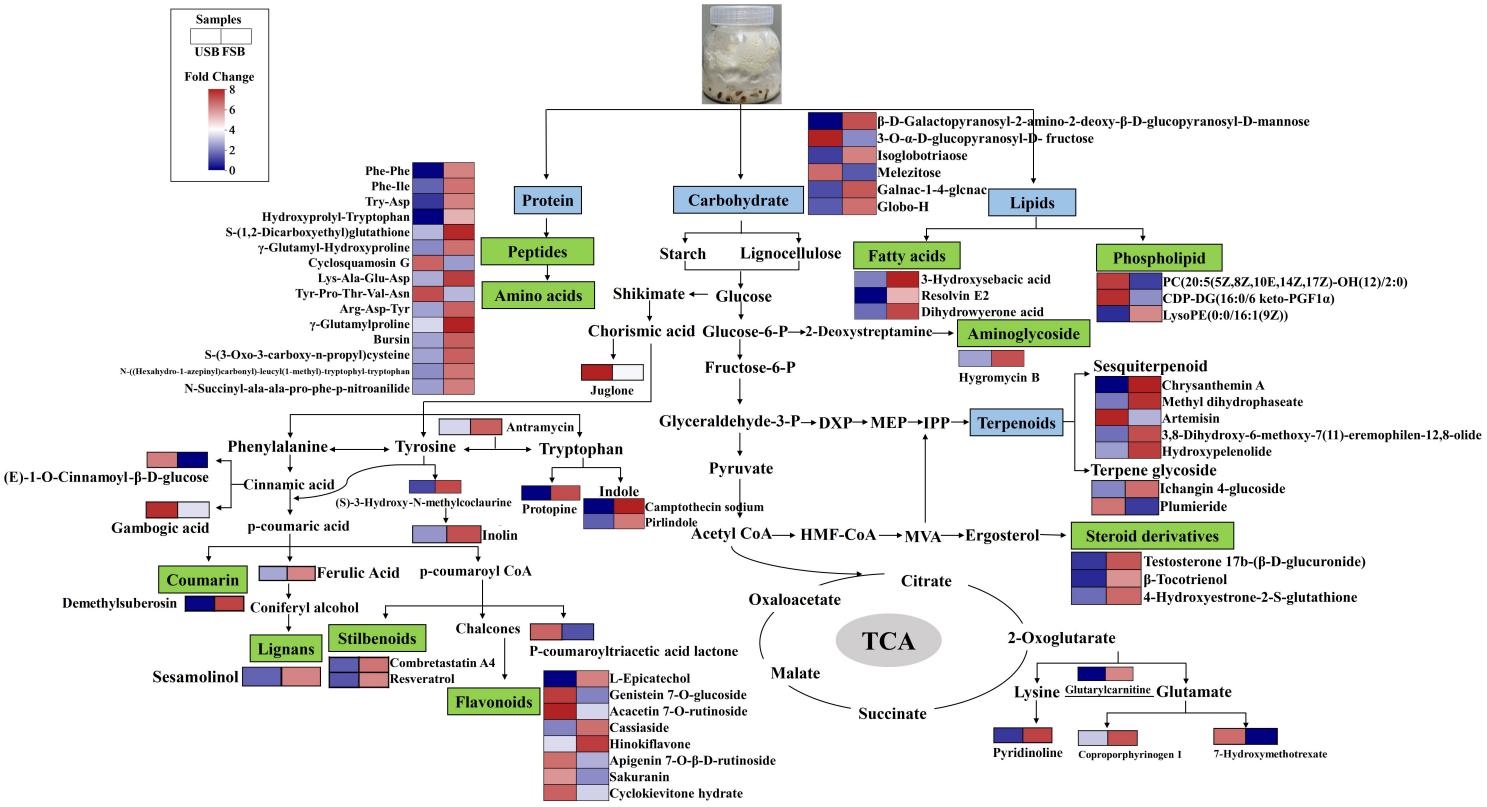
Biosynthetic pathway map of differential metabolites associated with antioxidant activity.

Based on the KEGG pathway analysis and metabolic data, a biological networking pathway map was created to show the dynamic and explain the non-volatile metabolite related to antioxidant activity of soybean after SSF with *P. ostreatus* (Figure 6). Soybean raw materials as well as macromolecules like proteins, carbohydrates, and lipids in this study could be broken down by *P. ostreatus* during fermentation through a variety of enzymatic activities (47). This generated substrates such as sugars, fatty acids, and amino acids that may be utilized in other cellular processes to form the characteristic metabolites that follow SSF. We only found small amounts of fatty acids in the differential metabolites after 33 days of fermentation, and neither monosaccharides nor amino acids were found (Figure 6). The aromatic amino acids phenylalanine, tyrosine, and tryptophan were synthesized through shikimate pathway, which are the precursors to the synthesis of phenolic compounds (127). Flavonoids, stilbenoids, coumarins, lignans and aromatic alkaloids were among the chemical substances whose overexpression in this study suggested that SSF could significantly increase the metabolism of the shikimic acid pathway and its downstream pathways (Figure 6). Camptothecin as an indole alkaloid, a modified monoterpene found in some plants (angiosperms) and endophytic fungi, is the source of the major anticancer medicine (123). This study is the first to demonstrate camptothecin can also be created by SSF of soybean with *P. ostreatus* (Figure 6). Through the EMP pathway, microorganisms convert glucose to pyruvate, which can then be transformed to a variety of carbohydrates and carbohydrate conjugates. Broad spectrum antibacterial agents were made from naturally occurring aminoglycoside antibiotics, which were mostly derived from bacteria (*Streptomyces* sp. and *Micromonospora* sp.) (128). An aminoglycoside antibiotic hygromycin B was identified in this study, demonstrating that this kind of compound can be synthesized and metabolized by mushroom mycelium (Figure 6). Terpenoids are the diverse family of natural products, and all terpenoids are derived from two 5-carbon precursors, dimethylallylpyrophosphate (DMAPP) and isopentenyl pyrophosphate (IPP), which are produced by the 2-C-methylerythritol phosphate (MEP) and mevalonate (MVA) pathways (129) (Figure 6). Among the components of fungi, steroids play a significant role, which are mostly metabolites of ergosterol. Numerous new metabolites of sterol and sterol derivatives from ergosterol were identified from fungal sources in recent years (130). Ergosterol conversion may be the source of the steroid derivatives in this investigation (Figure 6).

## 4 Conclusion

In this study, the utilization of *P. ostreatus* mycelium to enhance the quality of soybean products was explored for the first time. The investigation focused on nutritional ingredients, antioxidant activity and non-volatile metabolites during the SSF process. The results indicated a decrease in dietary fiber and starch content, while protein and lipid levels increased after SSF (*P* < 0.05). A significant increase in total phenolic content (from 4.62 to 20.72 mg GAE/g) was observed, along with increased antioxidant activities against intracellular reactive oxygen species (ROS), DPPH, and ABTS in lipopolysaccharide (LPS)-stimulated RAW 264.7 macrophages. Untargeted metabolomic analysis using UPLC-MS/MS revealed drastic differences in the metabolite profile of soybeans processed by SSF compared to the unfermented soybeans. A total of 154 differential metabolites covering 10 subclasses were detected after SSF, with amino acid and derivatives, carbohydrates, lipids, phenolic compounds, and terpenoids being the major classes of differential metabolites. The antioxidant activity of fermented soybeans might be associated with specific phenolic compounds, oligopeptides and free fatty acids, etc. SSF has been recognized as an effective bioprocess for enhancing the antioxidant activities of soybeans. The most abundant of these metabolites were phenolic compounds generated through the shikimic acid pathway. In the future, metabolomics could be used to explore the contribution of bioactive components to functional properties, thereby optimizing the production of bioactive components in fermented soy foods. Consequently, we suggest two applications for solid fermented soybean using mycelium from *P. ostreatus*: first, to improve the soybean's properties for the creation of meat analog, and second, as a bioreactor to produce bioactive compounds and medicines.

## Data Availability

The original contributions presented in the study are included in the article/Supplementary material, further inquiries can be directed to the corresponding author.
